# The Effect of Rituximab on Antiphospholipid Titers in Patients with Antiphospholipid Syndrome

**DOI:** 10.1055/s-0043-1770784

**Published:** 2023-07-05

**Authors:** Kimberley Youkhana, Hilary Heiling, Allison Deal, Stephan Moll

**Affiliations:** 1 Division of Hematology, Department of Medicine, University of North Carolina School of Medicine, Chapel Hill, North Carolina, United States; 2 Department of Biostatistics, Lineberger Comprehensive Cancer Center, University of North Carolina, Chapel Hill, North Carolina, United States

## Background

Rituximab in patients with antiphospholipid syndrome (APS) is mostly used for treating noncriteria APS manifestations such as thrombocytopenia, and is not a first- or second-line treatment for management of thrombosis. However, at times rituximab is used empirically as adjunct treatment in patients with recurrent thrombosis occurring in spite of anticoagulant therapy (i.e., anticoagulation failure) when combinations of increased anticoagulation intensity, addition of antiplatelet therapy, and reduction of all thrombotic risk factors fail to prevent recurrent thrombosis. 1 2 Knowledge of its effect on the naturally fluctuating antiphospholipid antibody (APLA) titers is, however, limited. We sought to determine the effect of rituximab on decreasing the magnitude and type of APLA in patients with thrombotic APS.

## Methods

This is a retrospective case series of adult patients (age ≥ 18) diagnosed with APS at the University of North Carolina Hospital inpatient and outpatient systems between August 2014 and August 2020. The electronic medical record encounter data were queried using the keyword “rituximab” and International Classification of Diseases 9th edition (ICD9/ICD10) codes for arterial and venous thromboses, APS, and catastrophic APS (CAPS). Venous thromboembolic events were defined as pulmonary embolism, deep vein thromboses of the upper and lower extremities, inferior vena cava, and renal veins. Arterial thromboses were considered if they affected major vessels. All thrombotic events were confirmed by chart review of radiology imaging reports. APLA titers were identified by current procedure terminology codes and considered positive if the lupus anticoagulant (LA) was positive (dilute Russell's viper venom time [DRVVT] ratio ≥1.20) in a DRVVT-based LA assay and/or hexagonal phospholipid neutralization (HPN) difference in an LA-aPTT-based assay >8 seconds, anticardiolipin (aCL) immunoglobulin G (IgG) >23 GPL, aCL IgM >10, and anti-β _2_ -glycoprotein (anti- β _2_ GPI) IgG or IgM >20 G or M units. The aCL and anti-β _2_ GPI positivity were arbitrarily categorized before data collection as low, medium, and high (< 40; 40–100; and >100 GPL, MPL, G units or M units, respectively).

“Time zero” was the start and stop dates of the first treatment course of rituximab. Negative or positive numbers indicated time before the start or after the stop dates, respectively. Laboratory observations 12 months before and after time zero were collected but censored after the start of the second treatment, if applicable. Titer changes are described.

## Results

Thirty-seven patients with APS were identified and 28 excluded for the following reasons: no available APLA titers ( *n* = 10); only one set of titers ( *n* = 10); no titers obtained within 12 months of treatment ( *n* = 5); only posttreatment titers available ( *n* = 3). Nine patients with thrombotic APS met the inclusion criteria for the primary analysis. Here are the associated diagnoses: APS only ( *n* = 4, including 1 patient with livedoid vasculopathy), CAPS ( *n* = 2), APS and immune thrombocytopenia ( *n* = 2), and APS and systemic lupus erythematosus ( *n* = 1). The medical records were reviewed for the indication for rituximab and use of concomitant treatment. At the time of treatment with rituximab, all patients were on therapeutic anticoagulation including antiplatelet therapy if they had arterial thrombotic events. All were exclusively treated with rituximab for thrombotic manifestations of APS, including the two patients with CAPS (ID#3 and 4) who also received therapeutic plasma exchange. The sample comprised women ( *n* = 8), median age of 47 years (40–79 years), Caucasian ( *n* = 6), black ( *n* = 2), and Hispanic/Latino ( *n* = 1). The following rituximab regimens were used: 375 mg/m ^2^ weekly ×4 doses ( *n* = 4), 1 g rituximab every 2 weeks ×2 doses ( *n* = 3), and 1 patient treated with both doses at two independent encounters.

**Fig. 1:**
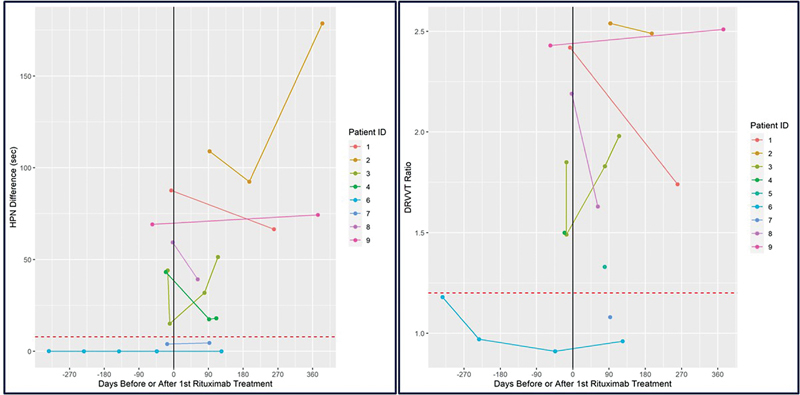
The changes in the HPN difference and the DRVTT ratio pre- and posttreatment with rituximab for each patient. Time zero is noted as “0” and marks the initiation of treatment. To the left of time zero is the pretreatment period and to the right is the posttreatment. Patients 3 and 4 were treated with therapeutic plasma exchange at the time of rituximab. DRVTT, dilute Russell viper venom time; HPN, hexagonal phospholipid neutralization.

Prior to treatment, 3 patients were triple APLA-positive, 2 were double, and 4 were single-positive based on the presence of a moderate or high positive aCL, anti-β _2_ GPI IgG titers, and/or positive LA. All 3 triple-positive patients remained triple-positive after treatment, 1 of 2 double-positive patients became single-positive, and 3 of 4 single-positive patients developed negative APLA tests. Pretreatment, 6 patients had a positive LA (5/6 positive HPN difference and 4/6 positive DRVVT ratio) ( Fig. 1 ).

In 2/4 patients, both the HPN and DRVVT ratio decreased, and in 2/4 both the HPN and DRVVT ratio increased. The mean decreases in HPN and DRVVT ratio were 58 seconds and 0.75, respectively. The mean increases in HPN and DRVVT ratio were 80 seconds and 0.625, respectively. The LA remained positive after treatment in all in whom the LA had been positive before treatment.

**Fig. 2:**
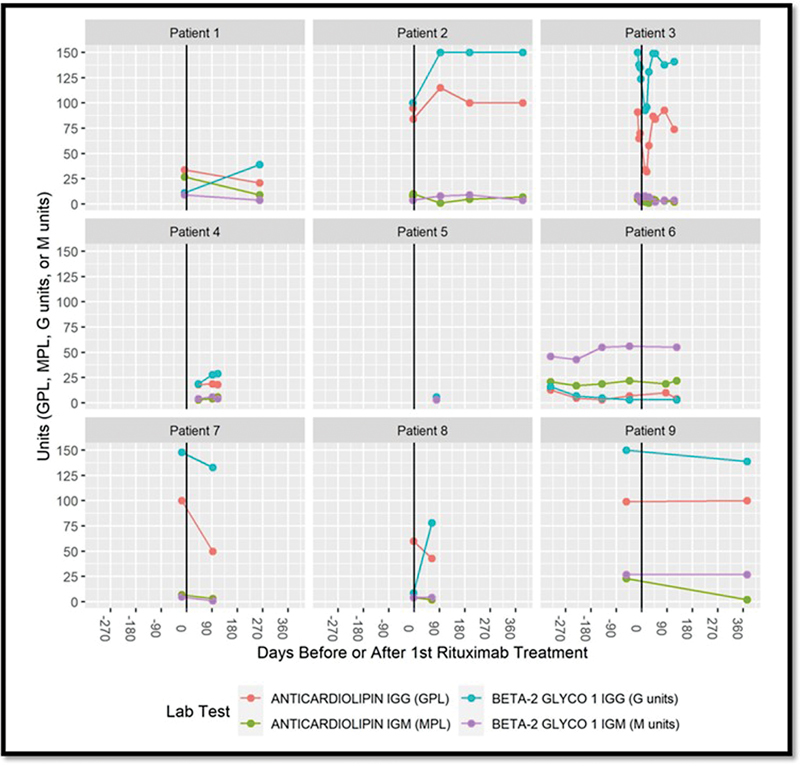
The changes in anticardiolipin and β-2-glycoprotein IgM and IgG titers pre- and posttreatment with rituximab. Time zero is noted as “0” and marks the initiation of treatment. To the left of time zero is the pretreatment period and to the right is the posttreatment. Patient 5 had a negative aCL and aβ2GPI titers measured 1,917 days before treatment—these pretreatment data points are not represented. Patients 3 and 4 were treated with therapeutic plasma exchange at the time of rituximab. aCL, anticardiolipin; IgG, immunoglobulin; IgM, immunoglobulin M.

The changes in aCL and anti-β _2_ GPI titers per patient are displayed in Fig. 2 . Among patients with titers in the medium range (5/6) and high range (1/6) and positive pretreatment aCL tests (6/9), all remained positive posttreatment: 3/6 remained in their pretreatment category, 1/6 increased from medium to high range, 1/6 decreased from medium to low, and 1/6 decreased from high to a medium titer range. Prior to treatment, all (3/9) aCL IgM-positive pretreatment titers were medium-range; posttreatment 2/3 became negative and 1/3 remained unchanged.

The aβ _2_ GPI IgG titers were abnormal in 6/9 patients. Pretreatment, 3/6 were low, 1/6 medium, and 2/6 in high-titer ranges. After treatment 3/6 remained unchanged, 2/6 increased from low to medium, 1/6 from medium to high, and 1/6 that had been negative pre-rituximab became positive. There were 2/9 pre- and posttreatment aβ2GPI IgM titers and these remained unchanged.

One of nine (1/9) patients had a recurrent thrombotic event within 1 year of rituximab treatment and died 2 years later from a spontaneous intracranial hemorrhage while on therapeutic anticoagulation and while a platelet count was 47,000 × 10 ^9^ /L.

## Discussion/Conclusion

We found that the use of rituximab in patients with APS did not meaningfully or consistently lower APLA titers. This finding is in keeping with the previous single-center prospective Erkan et al's study which showed no substantial change in the APLA profile of 19 APS patients—all patients who had positive APLA results at baseline had positive results at 24 and 52 weeks on follow-up testing. 3 In contrast, the retrospective study by Agmon-Levin et al showed that in 23 patients with APS who had pre- and post-rituximab APLA titer testing, there was a statistically significant decrease in APLA titers posttreatment in the group of 13 patients who had a clinical treatment response, but not in the group of 10 patients who did not have a clinical response. 4 Furthermore, our review of the literature of case reports and small case series shows that the effect of rituximab on APLA titers is inconsistent and equivocal—increasing, decreasing, or remaining unchanged in some patients. 5 6 7 8 9 10 11 12 13 14

While our study is limited by its small sample size due to the significant number of patients who had to be excluded because of nonavailability of APLA test results in the electronic medical record system, its retrospective nature, the inclusion of results obtained with various APL assays with the potential for inter-assay result variations, and the heterogeneity of the clinical phenotypes, it nonetheless serves as useful confirmatory data to the existing limited published literature that rituximab use in APS may not be a useful approach to lower APLA titers. Whether rituximab lowers future thrombosis risk in APS patients, particularly in those who have had an anticoagulation failure, has not been studied and our study does not provide an answer to this clinically important question. 8 However, the assumption that rituximab therapy as an adjunct to anticoagulation therapy might lead to a lower future thrombosis risk by lowering APLA titers is not supported by our study and may be better answered by prospective clinical trials. In summary, the results of our study, the 2013 Erkan et al study, and the limited case report and small case series literature dampen our enthusiasm to use empiric rituximab in patients with APS and recurrent venous thromboembolism to potentially lower APLA titers and, through that, the risk of recurrent thrombosis.
